# Characterizing Clinical Progression in Cognitively Unimpaired Older Individuals with Brain Amyloid: Results from the A4 Study

**DOI:** 10.14283/jpad.2024.123

**Published:** 2024-07-24

**Authors:** Dorene M. Rentz, P.B. Rosenberg, R.A. Sperling, M.C. Donohue, R. Raman, A. Liu, P.S. Aisen, A4 study team

**Affiliations:** 1 Departments of Neurology, Massachusetts General Hospital, Brigham and Women's Hospital, Harvard Medical School, 02115, Boston, MA, USA; 2 Memory and Alzheimer's Treatment Center, Division of Geriatric Psychiatry and Neuropsychiatry, Johns Hopkins Hospital, 21287, Baltimore, MD, USA; 3 Alzheimer's Therapeutic Research Institute, Keck School of Medicine, University of Southern California, 92121, San Diego, CA, USA; 4 Department of Neurology, Brigham and Women's Hospital, 60 Fenwood Road, 9016S, 02115, Boston, MA, USA

**Keywords:** Alzheimer prevention trials, clinical dementia rating scale, A4 study

## Abstract

**Background:**

Clinical Dementia Rating (CDR) global (CDR-G) and sum of box scores (CDR-SB) are commonly used as primary outcome variables to measure progression or treatment effects in symptomatic Alzheimer disease (AD) clinical trials.

**Objectives:**

We sought to determine whether the CDR is sensitive to change in pre-symptomatic AD and whether there are specific CDR boxes that are dynamic during the multi-year Anti-Amyloid in Asymptomatic Alzheimer's Disease (A4) secondary prevention study.

**Design:**

All participants entered the study with a CDR-G of 0. Box scores were examined individually and as composites of cognition (memory, orientation and judgment/problem solving) and function (community affairs and home/hobbies). A progression in box score was tabulated only when the change occurred at two consecutive visits.

**Setting:**

The A4 study took place at 67 sites in Australia, Canada, Japan and the United States.

**Participants:**

1,147 individuals, ages 65–85, were randomized to either placebo (n= 583) or solanezumab (n= 564). All participants received a baseline flobetapir PET scan, an annual CDR, and cognitive testing every 6 months with the Primary Alzheimer Cognitive Composite (PACC) over the course of 240 weeks.

**Measurements:**

Generalized estimating equations and generalized least square models were used to explore the modeled mean progression rate in the CDR-G, CDR-SB, individual CDR boxes, and CDR composite scores in the combined solanezumab and placebo groups. Models were refitted to explore the probability of CDR progression in centiloid tertiles of amyloid at baseline (< 46.1 CL, 46.1 to 77.2 CL, > 77.2 CL). All models included effects for age, education, APOEε4 carrier status, baseline amyloid with flobetapir PET, treatment, and time-by-treatment.

**Results:**

There were no statistical differences between the placebo or solanezumab groups in CDR-G, CDR-SB, specific CDR boxes or CDR composite scores over the course of the trial. Changes in judgment/problem solving were present at baseline and persisted over time, but progression on the CDR memory box and the CDR cognitive composite quickly predominated. Community affairs and home/hobbies showed little progression. Personal care remained stable. The probability of cognitive and functional progression in CDR boxes began either at the intermediate or advanced amyloid level (46.1 to 77.2 CL, > 77.2 CL), while amyloid at the lowest level (< 46.1 CL) showed relatively little CDR progression.

**Conclusions:**

The findings suggest that the CDR memory box and the CDR cognitive composite progressed over 240 weeks and were associated with intermediate and advanced stages of amyloid at baseline. Functional changes in community affairs and home/hobbies were relatively stable. These finding suggest that specific CDR box score changes may help refine our measurement of expected treatment effects in future AD prevention trials.

## Introduction

The Anti-Amyloid in Asymptomatic Alzheimer's Disease (A4) study was conducted to determine if treating individuals with amyloid burden prior to clinical symptoms could slow disease progression. While the results of the A4 study failed to show that solanezumab, an anti-monomeric amyloid antibody, slowed the progression of preclinical Alzheimer's disease (AD) as expected (1), the results of the A4 study provide a rich data source for understanding cognitive and functional decline at the preclinical stage.

The Clinical Dementia Rating Scale (CDR) (2) is a commonly used outcome variable to measure the natural progression of AD (3, 4, 5) and treatment effects in AD clinical trials (6, 7, 8). The advantage of the CDR is its ability to assess the impact of cognition on activities of daily living (ADLs) from an informant and clinician perspective. Cut-off scores for the CDR global (CDR-G) and sum of boxes (CDR-SB) have been derived from observational studies demonstrating good diagnostic accuracy for normal cognition, MCI and AD dementia (9). The CDR-SB has acceptable psychometric properties (4), provides a precise measure of cognitive dysfunction (3), and is thought to be an attractive primary outcome for use in AD clinical trials (4, 5, 10). The CDR-SB was recently used as a primary efficacy endpoint in the FDA approved clinical trial of lecanemab (8), a monoclonal antibody targeting A-Beta for the treatment of MCI and early AD. In the lecanemab study, the CDR-SB showed less progression in the treated group compared to placebo. In a study of donanemab (7), another monoclonal antibody targeting A-beta, the CDR-SB was used as a secondary outcome measure and showed a 39% lower risk of progressing to the next stage of disease compared to placebo. These studies demonstrate the usefulness of the CDR-G and CDR-SB in observational and clinical trials conducted at symptomatic stages of AD, but a more sensitive metric of specific CDR box change may be necessary as we move towards prevention in asymptomatic preclinical AD.

In the A4 study, all participants were required to be functionally normal at baseline (CDR-G=0) prior to randomization. While there were no significant differences in CDR-G or CDR-SB between A4 placebo and treatment groups (1), it is unknown which ADL functions declined and which, if any, ADL functions stayed stable over the length of the trial.

We sought to better understand whether the A4 study population of clinically unimpaired (CU) older adults with baseline evidence of brain amyloid burden was indeed at risk of significant cognitive and functional decline on the CDR. If so, what was the sequence of progression among different CDR domains (box scores) in contrast to the CDR-G and CDR-SB? Thus, we hypothesized: 1) The CDR memory box score will show the most progression and will change before all other box scores; and 2) that specific box scores associated with cognition, rather than function, will show the most progression for those with the most amyloid burden at baseline. We anticipate that knowing the specific sequence of CDR box progression in CU participants would potentially provide a more sensitive metric for measuring change or a potential treatment effect in future AD prevention trials.

## Methods

A4 methods have been previously published (1), and we summarize the relevant aspects here as follows.

### Participant Data

Participants came from the modified intent-to-treat population of the A4 study, which consisted of 1,147 individuals, ages 65–85. Participants were living independently without a diagnosis of Mild Cognitive Impairment (MCI) or dementia, and enrolled if they had a CDR-G score of 0 (range 0–3), Mini Mental State Exam (11) score of 25–30 (range 0–30) and a Weschler Memory Scale-Revised (12) Logical Memory Delayed Recall score of 6–18 (range 0–25). Persons with unstable medical conditions were excluded, although participants with stable hypertension, diabetes, hypercholesterolemia, mild-to-moderate small-vessel ischemic disease and other medical conditions were eligible. Participants were randomized 1:1 to either placebo (n= 583) or solanezumab (n= 564). All participants received a baseline flobetapir PET scan, an annual CDR, and cognitive testing every six months with the Primary Alzheimer Cognitive Composite (PACC) (13) over the course of 4.5 years. All participants were required to have a study partner who was familiar with their functioning and consented to participate in data collection.

### Study Conduct and Brief Description of the Intervention

The A4 study was an early intervention trial that aimed to slow disease progression in clinically unimpaired (CU) older adults who had elevated amyloid on PET at baseline. The trial was conducted at 67 sites including Australia, Canada, Japan and the United States. Solanezumab is an immunoglobulin GI monoclonal antibody that binds to the mid-domain of the A-beta monomer (14). Eli Lilly provided the trial drug and placebo. Treatment was administered as a monthly infusion. The double-blind phase of the trial was conducted over 240 weeks with a double-blind extension to 312 weeks to accommodate those participants whose last visit was delayed due to the COVID 19 pandemic hiatus.

### The Clinical Dementia Rating Scale (CDR)

The CDR (2) is a two-part structured interview conducted by a trained and certified clinician at each site. The CDR begins with a clinician interview of the A4 study partner; someone who can adequately rate baseline function and potential change over time. The second part of the CDR involves the clinician interview of the participant and consists of an objective assessment of memory recall, orientation and judgment/problem solving. Scoring is tabulated by the clinician based on the study partner interview and the clinician's objective assessment. The CDR-G rating is an ordinal scale ranked from 0–3 as follows: 0= no cognitive impairment, 0.5= mild cognitive impairment, 1= mild dementia, 2= moderate dementia and 3= severe dementia. Six domains are assessed on an ordinal scale in the CDR, consisting of memory, orientation, judgment/problem solving, community affairs, home and hobbies and personal care; these domains are termed ‘box scores'. The CDR-SB is tabulated by adding the ordinal score from each of the 6 domains to obtain a continuous variable, which has a range from 0 (no impairment in any of the boxes) to a maximum value of 18 (severe impairment with a score of 3 in all 6 domain boxes).

For this study, we were interested in the CDR-G and CDR-SB in relation to the individual domain CDR box scores and the derived composites of cognition (memory, orientation and judgment/problem solving) and function (community affairs and home/hobbies) as both an ordinal (0–1) and a continuous variable (0–18). We defined a ‘progressor' as someone who had a non-zero score in CDR-G or a change on an individual box score at two consecutive visits; all other participants were termed ‘non-progressors'.

### Brain amyloid

Brain amyloid was assessed with 18F-Florbetapir PET using a mean cortical imaging standardized uptake value ratio (SUVR) with a cerebellar reference as previously described (1). An SUVR threshold of 1.15 identified individuals with early amyloid accumulation. An SUVR of 1.10 to < 1.15 was considered elevated only when a visual read was considered positive by a two-reader consensus. SUVR's were converted to centiloids on a scale of 0 to 100.

### Statistical Methodology

Individual CDR box scores were modeled as a binary outcome (0 vs >0) using generalized estimating equations assuming unstructured correlation, and plots depict the modeled mean “progression rate”, or proportion with box scores >0, over time. Generalized least squares models assuming heterogeneous Toeplitz covariance were used to model CDR sum of boxes and composites. All models included effects for age, education, APOEε4 carrier status, baseline amyloid with flobetapir PET, treatment, and time-by-treatment. Time was modeled using natural cubic splines with two or three degrees of freedom guided by Akaike Information Criterion (15). Since there were no differences between treatment groups, summary plots show the trend averaging both groups. Models were refitted to explore the probability of progression for individual CDR box scores and composites within baseline amyloid PET centiloid tertiles (< 46.1 CL, 46.1 to 77.2 CL, > 77.2 CL).

## Results

### Cohort characteristics

Baseline characteristics of the cohort have been previously published (1) and are presented in Table 1. To summarize, participants had a mean age of ~72 years, had completed slightly > 16 years schooling, were 94% White, ~ 60% female, had a mean baseline MMSE of ~28, and 54.8% had at least 1 APOEε4 allele. The placebo and solanezumab groups were balanced on all baseline characteristics. There were no significant differences between treatment groups. The results of the CDR-G, CDR-SB, individual CDR boxes and CDR composites are presented here as an average of the two groups.

**Table 1:** Baseline characteristics of the A4 cohort

	Placebo (N=583)	Solanezumab (N=564)	Total (N=1147)
Age (y)	71.9 (5.0)	72.0 (4.7)	72.0 (4.8)
Female sex	352 (60.4%)	329 (58.3%)	681 (59.4%)
Education (y)	16.6 (2.9)	16.6 (2.7)	16.6 (2.8)
Racial categories			
White	549 (94.2%)	531 (94.1%)	1080 (94.2%)
Black or African American	15 (2.6%)	12 (2.1%)	27 (2.4%)
Asian	13 (2.2%)	11 (2.0%)	24 (2.1%)
American Indian or Alaskan Native	0 (0.0%)	1 (0.2%)	1 (0.1%)
More than one race	3 (0.5%)	5 (0.9%)	8 (0.7%)
Unknown or Not Reported	3 (0.5%)	4 (0.7%)	7 (0.6%)
Ethnicity			
Not Hispanic or Latino	560 (96.1%)	542 (96.1%)	1102 (96.1%)
Hispanic or Latino	18 (3.1%)	16 (2.8%)	34 (3.0%)
Unknown or Not reported	5 (0.9%)	6 (1.1%)	11 (1.0%)
Family history of dementia (parent or sibling)	449 (77.0%)	411 (72.9%)	860 (75.0%)
APOEε4	342 (58.7%)	333 (59.0%)	675 (58.8%)
FBP SUVr	1.3 (0.2)	1.3 (0.2)	1.3 (0.2)
FBP Centiloid	65.9 (32.1)	66.2 (33.5)	66.0 (32.8)
PACC	−0.0 (2.6)	0.0 (2.7)	0.0 (2.7)
LM Delayed Recall	12.7 (3.5)	12.6 (3.8)	12.6 (3.7)
MMSE	28.8 (1.2)	28.8 (1.3)	28.8 (1.3)
CFI Combined	3.6 (3.3)	4.0 (3.6)	3.8 (3.5)
ADL Partner	43.5 (2.6)	43.4 (2.7)	43.4 (2.6)
CDR-SB	0.0 (0.2)	0.1 (0.2)	0.1 (0.2)


Summaries include means and standard deviations for continuous variables and counts with percentages for binary or categorical variables.

### Trajectory of CDR-G and individual box scores

**Figure 1:**
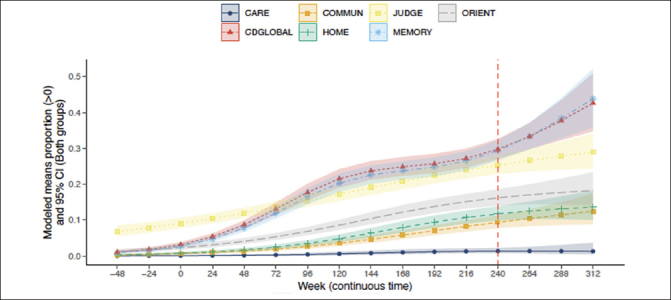
Trajectory of CDR-G and individual CDR box scores

The probabilities of CDR-G and specific box score progression after 240 weeks of treatment are presented in Figure 1. By the inclusion criteria, participants were required to have both CDR-G and a memory box score of 0 at baseline. In accordance with CDR scoring rules, participants were allowed to have a non-zero score at baseline in judgment and problem-solving and a small proportion of participants did. As can be seen in Figure 1, CDR-G and some specific box scores progressed over time in a substantial proportion of participants (higher CDR scores represent poorer cognition and function). The mean (95%CI) CDR-G at week 240 was 0.30 (0.27, 0.33). For the memory box score the mean was similar at 0.29 (0.27, 0.32) suggesting that the CDR-G is driven by memory changes. The judgment and problem-solving mean was 0.25 (0.23, 0.28), while means were smaller for orientation at 0.16 (0.14, 0.19], community affairs at 0.094 (0.077, 0.11], and very low in personal care at 0.014 [0.0081, 0.023]. While changes in judgment and problem solving were present in some individuals at baseline, over time, the memory box score continued to progress, preceding any changes in orientation, home and hobbies and community affairs. Personal care was unchanged throughout the course of the study.

#### Trajectory of CDR-G and CDR cognitive and functional composites

**Figure 2:**
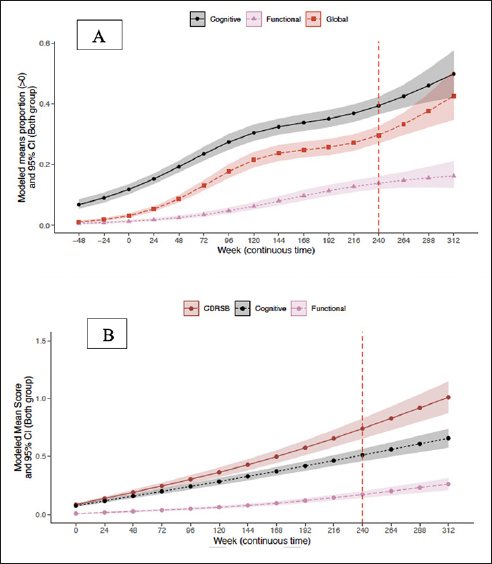
Trajectory of CDR-G (A) and CDR-SB (B) and cognitive and functional composites

CDR composites included three boxes that assessed cognitive function (memory, orientation, and judgment/problem solving) and two that assessed daily function (community affairs and home/hobbies). To compare CDR-G with composite outcomes, composites were treated as ordinal (0,1) and progression was tabulated only when two consecutive timepoints were achieved. The modeled means of CDR-G and CDR composites are presented in Figure 2A. Cognition declined before function, with CDR-G tracking as expected between the two. The probabilities (95% CI) of progression at week 240 for the CDR-G was 30% (27%, 33%), for the cognition composite was 39% (37%,42%); and the functional composite was 14% (12%, 16%).

### Trajectory of CDR-SB and cognitive and functional composites

To compare the CDR-SB to CDR composites of cognition and function, the CDR-SB and the CDR composites were treated as continuous (0–18). The modeled mean CDR composites are presented in Figure 2B. The CDR-SB showed the greatest progression with the cognitive composite tracking closely. Importantly, cognition progressed before function. The mean (95% CI) at week 240 for the CDR-SB was 0.74 (0.65, 0.83), for the cognition composite was 0.51 (0.46, 0.56) and the functional composite was 0.17 (0.14, 0.20).

### Trajectory of CDR-G and cognitive and functional composites by baseline amyloid

**Figure 3:**
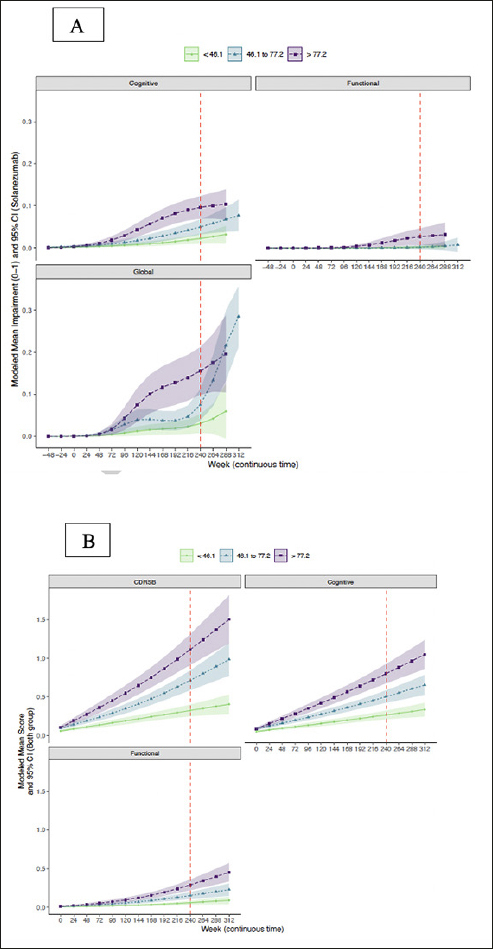
Trajectory of CDR-G (A) and CDR-SB (B) and cognitive and functional composites by amyloid tertiles

Trajectories of modeled mean CDR composite scores and CDR-G stratified by baseline amyloid tertile are shown in Figure 3A. It is apparent that the upper two tertiles (46.1–77.2 centiloids, >77.2 centiloids) had greater risk of progression on the CDR cognitive composite and CDR-G than the lowest tertile (<46.1 centiloids). Only the highest amyloid tertile was associated with functional progression. The mean (95% CI) at week 240 for the CDR-G at the lowest tertile was 0.02 (0.01, 0.03), for the intermediate tertile was 0.09 (0.06, 0.12) and the highest tertile was 0.14 (0.10, 0.17)). The mean (95% CI) cognitive composite at the lowest tertile was 0.02 (0.01 0.03), for the intermediate tertile was 0.05 (0.04, 0.07) and for the highest tertile was 0.08 (0.06, 0.10). The mean (95% CI) functional composite at the lowest tertile was 0.00 (0.00, 0.00), for the intermediate tertile was 0.00 (−0.00, 0.00) and for the highest tertile was 0.02 (0.01, 0.03).

### Trajectory of CDR-SB and cognitive and functional composites by baseline amyloid

Trajectories of the modeled mean CDR composite scores and CDR-SB stratified by baseline amyloid tertile are shown in Figure 3B. It is apparent that the upper two tertiles (46.1–77.2 centiloids, >77.2 centiloids) had greater risk of progression on the CDR-SB and the CDR cognitive composite in contrast to the lowest amyloid tertile (<46.1 centiloids). The modeled mean (95% CI) at week 240 for the CDR-SB at the lowest tertile was 0.33 (0.25, 0.40), for the intermediate tertile was 0.72 (0.59, 0.84) and the highest tertile was 1.11 (0.91, 1.33). The modeled mean (95% CI) of the cognitive composite at the lowest tertile was 0.27 (0.21, 0.32), for the intermediate tertile was 0.50 (0.42, 0.58) and for the highest tertile was 0.80 (0.67, 0.93). The modeled mean (95% CI) of the functional composite at the lowest tertile was 0.059 (0.035, 0.085), for the intermediate tertile was 0.15 (0.11, 0.20) and for the highest tertile was 0.29 (0.22, 0.36).

### Trajectory of CDR-G and individual CDR box scores by baseline amyloid

**Figure 4:**
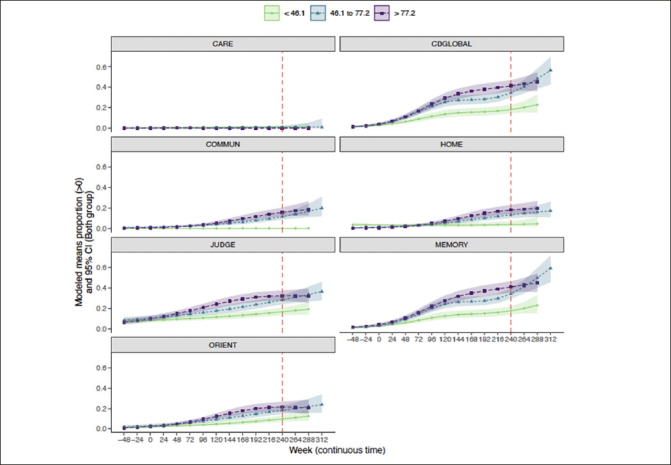
Trajectory of CDR-G and individual CDR box scores by amyloid tertiles

Similar results were observed when considering individual CDR boxes (Figure 4). Intermediate and higher baseline amyloid levels were associated with greater CDR progression, particularly in CDR-G, and CDR boxes of memory and judgment/problem solving. Home and hobbies and community affairs were less impacted by amyloid deposition. Personal care stayed stable at all levels of amyloid. The probability (95%CI) of progression at week 240 in CDR-G at the lowest tertile was 19% (15%, 23%), for the intermediate tertile was 35% (30%, 40%) and the highest tertile was 41% (37%, 47%). The probability (95% CI) of progression of the memory box score at the lowest tertile was 18% (14%, 22%), for the intermediate tertile was 34% (29%, 39%) and for the highest tertile was 41% (36%, 47%). The probability (95% CI) of progression of judgment/problem solving box score at the lowest tertile was 17% (13%, 21%), for the intermediate tertile was 28% (24%, 33%) and for the highest tertile was 32% (27%, 37%). The probability (95% CI) of progression of the orientation box score at the lowest tertile was 10% (7%, 13%), for the intermediate tertile was 18% (15%, 23%) and for the highest tertile was 21% (17%, 27%). The probability (95% CI) of progression of community affairs box score at the lowest tertile was 0% (0%, 0%), for the intermediate tertile was 11% (9%, 15%) and for the highest tertile was 16% (12%, 20%). The probability (95% CI) of progression of home/hobbies box score at the lowest tertile was 4% (2%, %7), for the intermediate tertile was 13% (10%, 17%) and for the highest tertile was 18% (14%, 23%). The probability (95% CI) of personal care at the lowest tertile was 1% (0%, 2%), for the intermediate tertile was 1% (0%, 2%) and for the highest tertile was 0% (0%, 0%).

## Discussion

The CDR-G and the CDR-SB are commonly used metrics for staging cognitive and functional changes in AD clinical trials. In the A4 secondary prevention study (1), we found that the risk of progression on the memory box score, as well as our derived cognitive composite of memory, orientation and judgment/problem solving provided a suitable metric of progression over 240 weeks, in contrast to the relative stability of community affairs and participation in home and hobbies. While the CDR-G also tracked with the memory box score, we found that the CDR cognitive composite was a better metric of progression than CDR-G and consistent with the expectation that CDR-G in this asymptomatic cohort was driven by memory changes. These findings suggest that the CDR-G, which encompasses CDR domains that are not changing, may be understating any real effect that could go undetected when using this global score. For the CDR-SB, the findings are a little more difficult to interpret because of the continuous nature of the scale. However, our conclusion is that cognitive/memory changes precede functional changes and that this is reflected in both CDR-SB and CDR-G outcomes.

In a prior study using the A4 screening data involving independent ADLs (IADLs) (16), four items stood out, including remembering appointments, finding one's belongings, following TV programs and remembering current events (16). While a broad range of IADLs were examined in this CU A4 cohort, the IADL items that were endorsed relied primarily on memory/cognitive changes and were associated with elevated cortical amyloid at screen. Another study that utilized the A4 screening data to examine items on the Cognitive Function Index, a measure of cognitive concerns (17), found specific memory endorsements in contrast to other concerns. Participants selected items that included an awareness of a memory decline over the past year and that they had seen a doctor for these memory concerns. Study partners endorsed the participants' repetition of questions and trouble following the news. These unique memory items were associated with elevated amyloid at screen.

These findings provide evidence that even when baseline cognitive test scores are normal, subtle memory changes are impacting ADL functioning in those with evidence of amyloid burden. The findings from our current analysis, along with these two A4 screening studies, suggest that choosing the CDR memory box or CDR cognitive composite, may improve our power to detect change in a secondary prevention trial, and should be considered in addition to more comprehensive measures such as a total test score or the CDR-G and CDR-SB.

It is well known from natural history studies that brain amyloid accumulation occurs 15–20 years prior to clinical symptoms and has been identified as an early biomarker of eventual progression to AD dementia (18). In the A4 study, participants were required to have a positive amyloid scan, predetermined by an SUVR threshold or, in borderline cases, consensus on a visual read. Interestingly, the probability of progression on the CDR memory box and cognitive composite score was associated with both intermediate and advanced levels of baseline amyloid, which essentially tracked together and was related to faster CDR progression. This observation that CDR memory box score changes are occurring, even at the intermediate amyloid level, supports the theory that a therapeutic intervention at earlier stages of amyloid (such as when memory starts to change) may be important for slowing disease progression and ultimately keeping individuals functional for longer periods of time.

In essence, the findings of this study suggest that specific CDR boxes may provide a more refined outcome measure targeted at specific daily functions that are vulnerable at the preclinical stage of AD. FDA guidance suggests that a clinically meaningful treatment effect in AD must have a positive effect on how an individual feels, functions or survives. In other words, does the treatment make a difference in the patient's ability to think, care for themselves and live independently (6). As we plan future prevention trials, the struggle to find the right outcome measure that incorporates the voices and expectations of patients and their families (19) is important. Refining the commonly used CDR to capture specific changes, without diluting the metric with functions that are stable, may provide a more nuanced method for determining a clinically relevant treatment effect at the preclinical stage of AD.

### Strengths and limitations

The A4 study recruited a large, well characterized cohort who were assessed with validated instruments of cognition and function. The timeframe of 240 weeks provided ample time for systematic observations. Retention was acceptable with 72% percent of the cohort having completed all assessments at the end of the study (1).

However, there are several major limitations to the interpretation of these results. In the absence of a drug treatment effect, we cannot determine which CDR boxes are most likely to change with drug treatment. The inferences we make are closer to a prospective observational study of participants with evidence of amyloid burden at baseline rather than a randomized controlled trial (RCT). Also, the timing of cognitive and functional changes using the CDR memory box or CDR composites may be tenuous given the annual administration of this measure. It is possible that the PACC, or specific items on the CFI or ADL assessments may provide a more precise measurement. Another major limitation is that participants were not representative of all older persons at risk for cognitive decline. The A4 cohort was healthier and had fewer co-morbidities than the general population. They were also highly educated, non-Hispanic Whites and thus, we are challenged to say whether the abovementioned inferences are applicable to nonwhite populations or those from other cultures or socioeconomic strata. We also know that the risk prediction added by APOE allelotype varies by ethnicity (20, 21) and that the risk of amyloid positivity differs in Black and Hispanic populations (22, 23), which limits our knowledge of how the probability of CDR progression will perform in these subgroups. Finally, this is a clinical trial sample, not a community-based sample, and the inclusion/exclusion criteria inevitably led to a cohort that is not highly representative of the general population.

Future work is needed to determine whether specific CDR box score changes may help refine our measurement of treatment effects in AD prevention trials and whether they operate in a similar fashion across a broader representative cohort from other countries and cultures.

## Conclusion

Amyloid positive, cognitively unimpaired older adults are indeed at significant risk of cognitive and functional decline. While the CDR-G and CDR-SB are good metrics for measuring AD progression at symptomatic stages of MCI and early AD dementia, our findings suggest that a more refined CDR outcome that targets daily functions impacted by memory, should be considered as an alternative method for detecting progression during a secondary prevention trial. We also found that an intermediate level of baseline amyloid was associated with the probability of CDR progression in memory and the CDR cognitive composite. These findings indicate that treating CDR memory changes at the earlier amyloid stage may help slow disease progression and facilitate maintenance of daily function. We conclude that the A4 trial is a rich resource for planning future prevention studies and suggest that trial designs might consider lowering the amyloid cut-off to an intermediate level and give consideration to utilizing a CDR memory box or CDR cognitive composite score as a primary outcome.

*Funding:* The A4 study was supported by a public-private-philanthropic partnership which included funding from the National Institute of Aging of the National Institutes of Health (R01 AG063689, U19AG010483 and U24AG057437), Eli Lilly (also the supplier of active medication and placebo), The Alzheimer's Association, the Accelerating Medicines Partnership through the Foundation for the National Institutes of Health, the GHR Foundation, the Davis Alzheimer Prevention Program, the Yugilbar Foundation, an anonymous foundation, and additional private donors to Brigham and Women's Hospital, with in-kind support from Avid Radiopharmaceuticals, Cogstate, Albert Einstein College of Medicine and the Foundation for Neurologic Diseases.

*Ethical Standards:* Approval from an institutional review board was obtained at each of the sites and all participants and their study partners provided written informed consent prior to data collection.

*Conflict of Interests:* DMR received salary support from the A4 study (R01 AG063689, U19AG010483 and U24AG057437) and has receive payment or honoraria from USC Institute on Methods and Protocols for Advancement of Clinical Trials in ADRD (IMPACT AD) course and External Advisory Boards from the University of California-Davis, Washington University, Boston University and Northwestern. She has also received travel support to ACTC meetings, to the University of California Advisory Board Meeting and the Washington University Advisory Board Meeting. PBR has received grant support from the National Institutes of Aging, Alzheimer's Clinical Trials Consortium, Richman Family Precision Medicine Center of Excellence on Alzheimer's Disease, Eisai, Functional Neuromodulation, and Lilly; honoraria from GLG, Leerink, Cerevel, Cerevance, Bioxcel, Sunovion, Acadia, Medalink, Novo Nordisk, Noble Insights, TwoLabs, Otsuka, Lundbeck, Acadia, MedaCorp, ExpertConnect, HMP Global, Sinaptica, Synaptogenix, and Neurology Week. RAS reports grant support from Eisai, and Eli Lilly and reported serving as a consultant for AbbVie, AC Immune, Alector, Bristol-Myers-Squibb, Ionis, Janssen, Genentech, Merck, Prothena, Roche, and Vaxxinity. MD reports that his spouse is a full-time employee of Janssen, and he has served as a consultant to Roche. RR has received research support from the National Institutes of Health (NIH), the Alzheimer's Association, American Heart Association, Eli Lilly and Eisai. AL has received research support from the National Institutes of Health (NIH), the Alzheimer's Association, American Heart Association, Eli Lilly and Eisai. PAS has received grants or contracts from the National Institutes of Health (NIH), Alzheimer's Association, Foundation for NIH (FNIH), Lilly, Janssen and Eisai and consulting fees from Merck, Biogen, AbbVie, Roche, and Immunobrain Checkpoint.
